# Pulmonary hypertension, right ventricular dysfunction and acute heart failure: a portentous consortium

**DOI:** 10.1186/cc14237

**Published:** 2015-03-16

**Authors:** HD Michalopoulou, HM Michalopoulou, P Stamatis, E Kostetsos, D Stamatis

**Affiliations:** 1 Metaxa Hospital, Athens, Greece

## Introduction

Pulmonary hypertension and right ventricular dysfunction (RVD) are frequently encountered in patients with acute heart failure. We sought a better understanding of the coupling between RVD and pulmonary hypertension in the setting of acute decompensated heart failure (ADHF) as it might improve the prognostic stratification and influence the survival rates.

## Methods

Echocardiography was performed in 329 patients with ADHF and right ventricular function was assessed by measuring the right ventricular fractional area, and a right ventricular ejection fraction (RVEF) <35% was taken as the cutoff value for RV systolic dysfunction. The systolic pulmonary pressure (PASP) was calculated from the tricuspid regurgitation signal applying the modified Bernoulli equation, and pulmonary hypertension was considered as PASP >35 mmHg. Based on the values of PASP and RVEF the study group was classified into four subgroups: group 1, normal PASP/preserved RVEF; group 2, high PASP/preserved RVEF; group 3, normal PASP/ low RVEF; group 4, high PASP/low RVEF. The primary endpoint was all-cause mortality. The median follow-up was 18 months. Survival analysis was performed according to the Cox regression method, adjusted for age, gender, LV function, estimated glomerular filtration rate, troponin I, hemoglobin, serum sodium and BNP levels.

## Results

Pulmonary hypertension was found in 78% of the patients (median PASP: 53 mmHg). As compared with the patients with normal PASP the patients with pulmonary hypertension were more likely to be in New York Heart Association functional class (NYHA) III or IV (86% vs. 49%, *P *< 0.001), had a lower RVEF (23 ± 9% vs. 32 ± 8%, *P *< 0.001), and had significantly higher BNP levels (280 ± 107 pg/ml vs. 540 ± 320 pg/ ml, *P *< 0.001). In a Cox model, compared with patients with normal right ventricular function and without pulmonary hypertension (group 1), the adjusted hazard ratio for mortality was 3.1 (95% CI: 1.6 to 4.2, *P *< 0.01) in group 2, 0.3 (95% CI: 0.2 to 1.9, *P *= 0.3) in group 3 and 4.2 (95% CI: 1.9 to 6.1, *P *< 0.001) in group 4.

## Conclusion

Among ADHF patients, the coupling of pulmonary hypertension and RVD carries an incremental risk, having a portentous impact on the survival rate.

